# Young children experience little emotional burden during invasive procedures in asthma research

**DOI:** 10.1007/s00431-018-3265-0

**Published:** 2018-11-03

**Authors:** Anne M. Padding, Niels W. Rutjes, Simone Hashimoto, Amit Vos, Mira S. Staphorst, Wim M. C. van Aalderen, Marc P. van der Schee

**Affiliations:** 1 Pediatric Respiratory Medicine, Emma Children’s Hospital, Amsterdam UMC, Amsterdam, The Netherlands; 2 Department of Pediatrics, Amsterdam UMC, de Boelelaan 1112, NL-1081 HV Amsterdam, The Netherlands; 3 Amit Vos Child Psychology, Amsterdam, The Netherlands

**Keywords:** Children, Asthma, Clinical research, Invasive procedures, Emotional burden, Puppetry

## Abstract

Research in children should strike the right balance between protecting underage study subjects and advancing the medical field. This study gives insight into the emotional burden that common invasive research procedures in asthma research have on young children, both from the child and parent perspective. Puppetry was used to stimulate children (age 5–6 years) to explain their emotional burden prior to and after the research procedures. We operationalised emotional burden as willingness to participate in future research and reluctance towards participation. Parents filled out a questionnaire on this topic. Symptomatic patients as well as healthy controls were analysed. Forty-one children were included. Children’s anticipatory fear for future research showed a clear decrease of 0.7 ± 1.6 on a 5-point Likert scale as a consequence of participation (*p* = 0.02). Sixty percent of all participating children explicitly indicated willingness to undergo identical research procedures again. Children uninformed by their parents about the venipuncture were significantly more reluctant to the venipuncture after the procedure (*p* < 0.01), compared to children who had been informed (4.0 ± 0.9 resp. 2.8 ± 1.2).

*Conclusion*: This study suggests that the emotional burden of participation in asthma research for underage children can be prevented when they are properly informed and decreases as a consequence of participations. We believe increased emphasis should be placed on informing children and evaluating the emotional impact of research to help caretakers and research ethics committees make informed decisions about participation of children in medical research.

**Table:** 

**What is Known:** • *Medical professionals and parents are likely to overestimate children’s discomfort undergoing (invasive) research procedures.* • *Two thirds of children (age 6–18 years) participating in medical research indicated that they would participate in the same research study again.*	
**What is New:** • *Pre-school children experience little emotional burden during invasive procedures in asthma research.* • *Proper communication about (invasive) research procedures in pre-school children helps to reduce the anticipatory fear of these procedures in the future.*

**Electronic supplementary material:**

The online version of this article (10.1007/s00431-018-3265-0) contains supplementary material, which is available to authorized users.

## Introduction

Despite widespread awareness that children are not small adults, evaluation and implementation of new technological and pharmacological innovations are often limited in children [1, 2]. As a consequence, uncertainty about safety and effectiveness leads to delayed implementation of these innovations in children. Limited knowledge of the burden and effect of participating in research procedures contributes to this lack of evidence-based medicine.

The number of clinical research projects involving children has been increasing over the last 10 years [3]. This increase has caused concern; research in children should strike the right balance between protecting underage study subjects and advancing the medical field to the benefit of all children.

New Dutch legislation (Art. 3 J. WMO 2017) requires researchers to describe the expected level of the burden that comes with participating in their research. Strikingly, very limited evidence is available on the attitudes of children towards invasive research procedures, particularly in young children (≤ 6 years of age). The evaluation of the experienced burden is now primarily based on intuition and experiences of the Research Ethics Committees, with no data to support whether this represents children’s views and experiences adequately. Subsequently, no validated methods are available for evaluating the emotional burden of research procedures on pre-school children.

This study aims to provide insight into the emotional burden that common invasive research procedures in asthma research have on young children and their parents. We hypothesise this experienced burden to differ between subjects with and without a clinical indication to undergo such procedures. We assessed differences in prospective and retrospective reluctance towards participating in future medical research, willingness to participate in future medical research and disappointment or an experience better than anticipated.

## Methods

We evaluated in 41 children (5–6 years) participating in the EUROPA study [4] anticipatory fear prior to research procedures as well as resentment after completion. Children underwent multiple research procedures including pulmonary function testing, a nasopharyngeal brush and venipuncture. Patients were divided into two groups: a symptomatic and a control group. Children in the control group had been symptomatic (wheezing/dyspnoea), while children in the control group are asymptomatic. The selected age range was based on children being diagnosed with asthma and being able to perform a pulmonary function test.

We evaluated the children via a playful interaction with puppets which was structured by a child psychologist, because questionnaires might be an unreliable way to measure anticipatory fear and resentment in young children [5–7]. Puppets were used to stimulate the child to answer the questions. The researcher asked questions both prior to and after undergoing the research procedure as if the puppet was asking, children were stimulated to tell the puppets what to expect. Parents were asked not to help their child. This approach circumvents the need for children to use self-reflection, which is still limited at this age. During these interactions children were explicitly asked if they felt the procedure was scary (Appendix 1). Answers to questions were scored by the interviewer on a 3-point visual analogue scale (not scared-very scared) and was later converted to a 5-point scale. All children were interviewed by the same researcher; this researcher was not blinded to children being cases or controls. Cognitive development was considered normal for all participating children. All children went to school and were able to perform a pulmonary function test.

Parents were asked to fill out a questionnaire scoring the reluctance to have their child participate in the study. They scored their answers on a similar scale as the researcher (Appendix 2). Additionally, willingness to participate in future research, the underlying motivations and the consequences of (not) informing a child about venipuncture prior to the visit on experienced burden were explored. The questionnaire was specifically created for this research study, because of a lack of validated instruments for this purpose.

### Answers were entered into SPSS statistics 23 software for analysis

A McNemar’s test was used to analyse the willingness to participate in future research prior to and after the research visit. Differences between cases and controls were analysed with a chi-square test. A Mann-Whitney *U* test and Wilcoxon signed-rank were used where appropriate to analyse the differences in parent’s and child’s mean reluctance towards medical research and venipuncture. A difference of 10% (> 0.5 point on a 5-point scale) or more was considered to be meaningful. For all of the analyses above, *p* < 0.05 was considered statistically significant.

## Results

We evaluated 41 parent-child couples, of which 6 couples did not provide answers to the post-intervention assessment (Table 1). Across all children, reluctance to undergo the research procedures, reported after the procedures, was 0.7 ± 1.6 points lower than reluctance reported prior to the procedure (*p* = 0.02). Similarly, children’s reluctance to undergo venipuncture decreased 1.0 ± 1.6 points (*p* < 0.01). Sixty percent of all participating children explicitly indicated willingness to undergo the identical research procedures again, even despite some parents being more reluctant to participate again (Table 2).

**Table 1:** Subject characteristics

	Cases	Controls
N	19	22
Gender (% boy)	12 (63)	12 (54)
Mean age in years, (SD)	5.8 (0.1)	5.8 (0.1)
Father completed higher education, no. (%)	12 (63)	19 (86)
Mother completed higher education, no. (%)	14 (74)	17 (77)
First degree relative with studied disease, no. (%)	10 (53)	2 (9)*
Hospital admission (%)	5 (26)	4 (18)
Hospital admission last year (%)	1 (5)	2 (9)
Missing retrospective values	5	1

*p value < 0.01

**Table 2:** Perspective on participation, cases compared to controls

	Prior	After
Parental reluctance to participate (1–5 scale)		
Cases	1.79 (0.98)	2.07 (1.39)
Controls	1.19 (0.40)	1.67 (1.07)*
Child reluctance to participate (1–5 scale)		
Cases	3.93 (1.06)	2.86 (1.21)*
Controls	3.25 (1.12)	2.86 (1.07)
Parental reluctance to venipuncture (1–5 scale)		
Cases	2.57 (1.09)	2.21 (1.53)
Controls	2.23 (1.09)	1.90 (1.0)
Child reluctance to venipuncture (1–5 scale)		
Cases	4.03 (1.11)	3.21 (1.53)*
Controls	3.65 (1.39)	2.94 (1.04)*
Parental willingness to participate in future research		
Cases (%)	64	71
Controls (%)	91	100
Willingness to participate in future research; children		
Cases (%)	N.A.	64
Controls (%)	N.A.	57

All values are mean (SD). *p value < 0.05 for prior compared to after

Parents of children with respiratory complaints were more reluctant to participate prior to the visit than parents of controls, respectively 1.8 ± 1.0 and 1.2 ± 0.4 (*p* < 0.01), which may be explained by prior hospital experiences. Twenty-two percent of children were not informed in advance by their parents about the venipuncture (despite explicit instructions), they were significantly more reluctant to the venipuncture after the visit (*p* = 0.02), compared to children who had been informed in advance (4.0 ± 0.9 and 2.8 ± 1.2, respectively).

The primary reasons for participation were altruistic (“research is important”/“to help other children”) in 66% of parents, followed by having a family member with the researched illness (34%). Thirty-nine percent of parents regarded the emotional burden to their child as their main concern. Children reported most anxiety around the venipuncture.

## Discussion and conclusion

Our results indicate that undergoing clinical research procedures does not increase reluctance to undergo future medical procedures. The anticipatory anxiety experienced by many children is likely a consequence of having a limited understanding of the nature and impact of hospital procedures. This is underpinned by our observation that informing children about the venipuncture has a positive impact on how they experience the procedure.

It is important to note that all of the parents of children in the control group indicated willingness to undergo these invasive research procedures again. Parents of children with respiratory complaints being more reluctant towards participating in medical research could be caused by previous experiences in hospitals or anticipatory parental fear of establishing an illness in their child.

Staphorst et al. [8, 9] have conducted research in children with a mean age of 11.9 years and 10.6 years, respectively, evaluating experienced burden of participation in medical research and have provided information on how to reduce this burden. They reported corresponding results, showing two thirds of children willing to participate in a similar research study. We extend these data, finding similar results, with our study being the first to examine the perspectives of younger, pre-school children and also the first study to show the importance of informing the child properly prior to the research procedures.

A strength of this study is the adjustment of our interviewing techniques to match the requirements of children by using a standardised puppetry play for the child. Even though this means the answers of children were interpreted by the interviewer, we believe this approach carries an implicit strength as it allows for proper adjustment of the question to the level of understanding of the child and prevents socially desirable answers. To further standardise this, all interviews were conducted by the same researcher.

A limitation of this study is its inability to discern the relative impact of the various procedures on the experience of the child.

Approximately 25% of all subjects participating in the EUROPA study participated in this research study. This is because of a limited timeframe (2 months) in which the researcher was available to perform the interviews. All subjects in this 2-month period participated in our research study. We do not expect this to have resulted in a selection bias.

As the interviewer was not blinded to whether patients were cases or controls this may have biased our results. Blinding the researcher to whether patients were cases or controls would be an improvement in a future study design. Parents were in the same room with their children; when the children were being interviewed, this may have caused alteration in the answers to the parent questionnaire. Separation of parent and child could prevent this latter bias, but is not desirable in children this age.

Contrary to common belief, the experienced emotional burden of asthma research is limited, even if this includes invasive procedures such as venipuncture. Clear prerequisite for this is to adequately inform the children. These results support the acceptability of children participating in medical research and can guide Research Ethics Committees in evaluation of the burden associated with participating in medical research, stimulating evidence-based medicine. Our study can also help parents and children who are approached to participate in future clinical research and will provide them with additional information on which they can base their decision to participate and manage their expectations. It will help parents to be able to prepare their child for participation.

We would like to stimulate fellow paediatric researchers to consider including a routine assessment of the experienced burden of research procedure on participating children and their parents. This way more data can be collected and disseminated, leading to improved evidence for the acceptability of children participating in medical research.

Proper communication shows to be important and further research could help us identify instruments, like cartoons or puppets, to communicate clear age-appropriate information to both parents and child.

## Electronic supplementary material

Appendix 1 (DOCX 14 kb)

Appendix 2 (DOCX 14 kb)

## Abbreviations

EUROPA study: Early Unbiased Risk-Assessment of Paediatric Asthma study
WMO: Wet Maatschappelijke ondersteuning (Dutch Social/Medical law)
