# Supplementary material for: Young children experience little emotional burden during invasive procedures in asthma research
Source: Eur J Pediatr. 2018 Nov 3;178(2):207–11. doi: 10.1007/s00431-018-3265-0 (PMC6339656; doi:10.1007/s00431-018-3265-0)
Supplement: Supplementary file 1 — (DOCX 14 kb) [file 431_2018_3265_MOESM1_ESM.docx]

**Appendix 1. Questions for the child.**

Questions for the child prior to research visit:

- Do you know why you’re here today? What would you tell the puppet about today? (open-ended response)
- What part of today will the puppet be most afraid of? (open-ended response)
- Do you feel bad about missing school today? (yes/no response)
- How scary would you tell the puppet today will be? (three-point scale)
- How scary would you tell the puppet the venepuncture will be? (three-point scale)

Questions for the child after the research visit:

- What would you tell the puppet about today? (three-point scale, worse/same/better than expected )
- What would you tell the puppet about the best part of today? (open-ended response)
- What would you tell the puppet about the worst part of today? (open-ended response)
- How scary would you tell the puppet today will be? (three-point scale)
- How scary would you tell the puppet the venepuncture will be? (three-point scale)
- Would you be willing (or would you tell the puppet) to participate in a day like this in the future? (yes/no response)
