# Supplementary material for: Young children experience little emotional burden during invasive procedures in asthma research
Source: Eur J Pediatr. 2018 Nov 3;178(2):207–11. doi: 10.1007/s00431-018-3265-0 (PMC6339656; doi:10.1007/s00431-018-3265-0)
Supplement: Supplementary file 2 — (DOCX 14 kb) [file 431_2018_3265_MOESM2_ESM.docx]

**Appendix 2. Parent questionnaire.**

Questions for the parent prior to research visit:

- Why did you decide to participate today? (open-ended response)
- Did you discuss participation with your child? (yes/no response)
- What are your concerns/disadvantages about participating today? (open-ended response)
- In what amount are you reluctant to the visit today? (five-point scale for reluctance)
- In what amount are you reluctant to the blood withdrawal today? (five-point scale for reluctance)
- Would you be willing to participate in medical research again in the future? (yes/no response)

Questions for the parent after the research visit:

- How did you experience the research visit today? (five-point scale for experience)
- In what amount would you be reluctant to a similar research visit in the future? (five-point scale for reluctance)
- In what amount would you be reluctant to the drawing of blood in the future? (five-point scale for reluctance)
- What could be improved about the visit today? (open-ended response)
- Would you be willing to participate in medical research again in the future? (yes/no response)
- Do you think there should be a monetary reward for participation in medical research with children? (yes/no response)
